# Phenalen-1-one-Mediated Antimicrobial Photodynamic Therapy: Antimicrobial Efficacy in a Periodontal Biofilm Model and Flow Cytometric Evaluation of Cytoplasmic Membrane Damage

**DOI:** 10.3389/fmicb.2018.00688

**Published:** 2018-04-06

**Authors:** Fabian Cieplik, Viktoria-Sophia Steinwachs, Denise Muehler, Karl-Anton Hiller, Thomas Thurnheer, Georgios N. Belibasakis, Wolfgang Buchalla, Tim Maisch

**Affiliations:** ^1^Department of Conservative Dentistry and Periodontology, University Medical Center Regensburg, Regensburg, Germany; ^2^Division of Oral Microbiology and Immunology, Clinic of Preventive Dentistry, Periodontology and Cariology, Center of Dental Medicine, University of Zurich, Zurich, Switzerland; ^3^Division of Oral Diseases, Department of Dental Medicine, Karolinska Institutet, Solna, Sweden; ^4^Department of Dermatology, University Medical Center Regensburg, Regensburg, Germany

**Keywords:** biofilm, periodontal, photodynamic, phenalen-1-one, chlorhexidine, flow cytometry, propidium iodide, cytoplasmic membrane

## Abstract

In light of increasing resistance toward conventional antibiotics and antiseptics, antimicrobial photodynamic therapy (aPDT) may be a valuable alternative, especially for use in dentistry. In this regard, photosensitizers (PS) based on a phenalen-1-one structure seem to be especially favorable due to their high singlet oxygen quantum yield. However, the actual target structures of phenalen-1-one-mediated aPDT are still unclear. The aim of the present study was to investigate the antimicrobial efficacy of aPDT mediated by phenalen-1-one derivatives SAPYR and SAGUA for inactivation of a polymicrobial biofilm consisting of three putative periodontal pathogens *in vitro* and to get first insights in the mechanism of action of phenalen-1-one-mediated aPDT by assessing damage of cytoplasmic membranes. aPDT with SAPYR exhibited identical antimicrobial efficacy as compared to chlorhexidine (CHX) [4.4–6.1 log_10_ reduction of colony forming units (CFUs) depending on bacterial species] while aPDT with SAGUA was less effective (2.0–2.8 log_10_). Flow cytometric analysis combined with propidium iodide (PI) staining revealed no damage of cytoplasmic membranes after aPDT with both phenalen-1-one derivatives, which was confirmed by spectroscopic measurements for release of nucleic acids after treatment. Spectrophotometric PS-uptake measurements showed no uptake of SAPYR by bacterial cells. Despite the inability to pinpoint the actual target of phenalen-1-one-mediated aPDT, this study shows the high antimicrobial potential of phenalen-1-on mediated aPDT (especially when using SAPYR) and represents a first step for getting insights in the mechanism and damage patterns of aPDT with this class of PS.

## Introduction

Periodontal disease is among the most prevalent diseases worldwide according to the most recent Global Burden of Disease study (1990–2015) affecting more than 537 million adults (GBD 2015 Disease and Injury Incidence and Prevalence Collaborators, 2016) and it is known to be the major cause for tooth loss (Tonetti et al., 2017). For patients suffering from aggressive periodontitis or severe or refractory forms of chronic periodontitis, it is common clinical practice to prescribe antibiotics (i.e., amoxicillin and metronidazole) adjunctively to subgingival debridement (van Winkelhoff et al., 1996; Zandbergen et al., 2013). However, this attracts critical voices nowadays (Preus et al., 2014), as more periodontal pathogens increasingly exhibit resistance toward these antibiotics (Rams et al., 2014). Rams et al. (2014) tested subgingival biofilm specimens from 400 adult patients suffering from chronic periodontitis in the United States and found one or more species resistant against either amoxicillin or metronidazole in approximately 43 or 30% of the patients, while 15% harbored subgingival periodontal pathogens resistant to both amoxicillin and metronidazole. Furthermore, recently first strains have been reported that exhibit resistance against the oral gold-standard antiseptic chlorhexidine (CHX) *in vitro* (Horner et al., 2012; Kitagawa et al., 2016), which may even be linked to multi-drug resistance (Saleem et al., 2016). Therefore, it has been recommended that its use should be limited to the applications “with a clear patient benefit” (i.e., in intensive care patients) in order to reduce the risk of inducing acquired resistances to CHX in pathogens (Kampf, 2016).

In the recent 2016 Review on Antimicrobial Resistance, the alarming scenario was built that the number of deaths due to antimicrobial resistance could increase to 10 million per year in 2050, potentially leading to cumulative economical costs of 100 trillion USD, if no action is taken immediately (O’Neill, 2016). Especially in dentistry, where usually no life-threatening diseases need to be treated, it should be a major goal to restrict the use of conventional antimicrobials and promote research on novel antimicrobial approaches with less risk of inducing resistance. In this light, antimicrobial photodynamic therapy (aPDT) may be a promising alternative (Gursoy et al., 2013). The principle of aPDT is based on irradiation of a *per se* non-toxic dye, the so-called photosensitizer (PS), by light of an appropriate wavelength in the presence of molecular oxygen. The absorption of light by the PS-molecule leads to its transition to an excited state, whereupon there are two mechanisms to let the PS regain its ground state: In the type I mechanism, charge is transferred to a substrate or molecular oxygen resulting in generation of reactive oxygen species (ROS) like superoxide anions ($O_{2}^{•–}$), hydrogen peroxide (H_2_O_2_), or free hydroxyl radicals (HO^∙^). In type II mechanism, energy (but no charge) is transferred directly to molecular oxygen, whereby singlet oxygen (^1^O_2_) emerges, which is regarded as the most effective ROS. Both processes can occur simultaneously in a PS, while the singlet oxygen quantum yield Φ_Δ_ describes the proportion of type II mechanism (Maisch et al., 2007; Cieplik et al., 2014; Wainwright et al., 2017).

In general, aPDT-mediated killing of bacteria is based on unselective oxidative damage of bacterial proteins, lipids and nucleic acids (Almeida et al., 2015). Thereby, oxidative damage of two major cellular components, i.e., cytoplasmic membranes and DNA, has been proposed (Alves et al., 2014; Almeida et al., 2015), but is still a matter of debate since the actual target sites mostly hinge on the respective cellular localization of the PS, which in turn depends on its chemical structure (molecular weight, charge, lipophilicity) and the cell wall characteristics of the targeted bacteria (Alves et al., 2014; Kashef and Hamblin, 2017). For damage of DNA, a PS has to build up high intracellular concentrations (Cadet et al., 2010), wherefore it seems reasonable that external structures like cell walls and cytoplasmic membranes are first-line targets and nucleic acids may only be affected when these external structures have already been destroyed (Almeida et al., 2015). Accordingly, classic studies show that the extremophile bacterium *Deinococcus radiodurans* can be easily inactivated by aPDT despite its highly efficient DNA repair mechanisms (Schäfer et al., 1998; Nitzan and Ashkenazi, 1999).

Phenalen-1-one-mediated aPDT has already shown its high antimicrobial potential and may be particularly advantageous since PS based on a phenalen-1-one structure nearly quantitatively react according to the type II mechanism (singlet oxygen quantum yields Φ_Δ_ ≥ 0.85) (Cieplik et al., 2013, 2015, 2016, 2018; Späth et al., 2014; Tabenski et al., 2016). However, target structures of aPDT with this class of PS are still unknown. Therefore, the aim of the present study was to combine traditional culture techniques and flow cytometry in order to (I) investigate the antimicrobial efficacy of phenalen-1-one-mediated aPDT for inactivation of a polymicrobial biofilm cultured from three putative periodontal pathogens *in vitro* and (II) to get first insights in its mechanism of action by assessing damage of cytoplasmic membranes.

## Materials and Methods

### Chemicals and Light Source

SAPYR (1-((1-Oxo-1H-phenalen-2-yl)methyl)-pyridinium chlo-ride) and SAGUA (1-((1-Oxo-1H-phenalen-2-yl)methyl)-1-methyl-guanidinium chloride) were provided by TriOptoTec GmbH (Regensburg, Germany), synthesized according to published protocols yielding a purity ≥99% (Cieplik et al., 2013; Späth et al., 2014; Tabenski et al., 2016). PS-suspensions were freshly prepared for the experiments in a concentration of 100 μM (dissolved in distilled water) and stored in the dark at 4°C for no longer than 2 weeks. For irradiation of both PS, a gas-discharge lamp (Waldmann PIB 3000; Waldmann Medizintechnik, Villingen-Schwenningen, Germany) was used (λ_em_ 380–600 nm). Irradiance was adjusted to 50 mW/cm^2^ at sample-level and irradiation was for a period of 10 min, resulting in an energy dose of 30 J/cm^2^.

Chlorhexidine digluconate (CHX) was obtained from the pharmacy department of the University Medical Center Regensburg in concentrations of 0.06 and 0.2% (w/v; dissolved in distilled water). Metronidazole (MET; Sigma-Aldrich, St. Louis, MO, United States) was diluted in distilled water yielding concentrations of 15 and 50 μg/mL.

**FIGURE 1:**
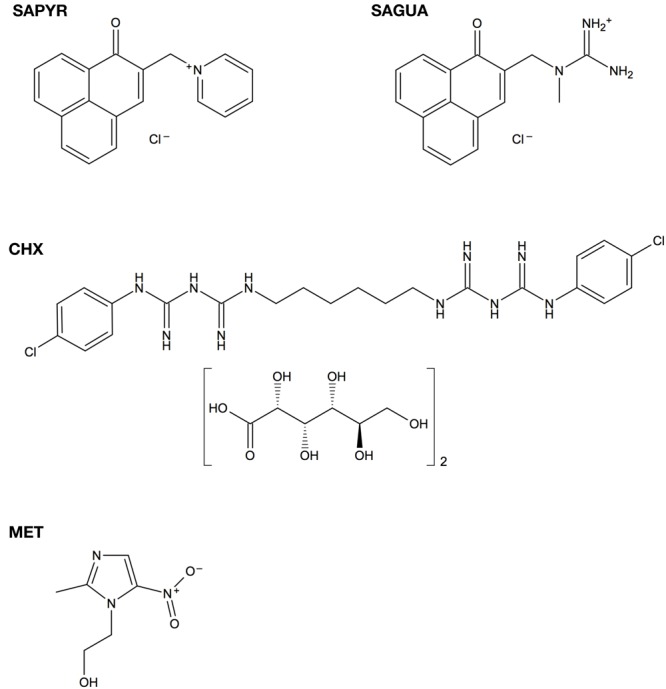
Chemical structures of SAPYR, SAGUA, chlorhexidine digluconate (CHX), and metronidazole (MET).

**Figure 1** shows the chemical structures of SAPYR, SAGUA, CHX, and MET.

### Bacterial Culture and Biofilm Formation

Three reference strains, *Actinomyces naeslundii* (DSM-43013), *Fusobacterium nucleatum* (DSM-20482), and *Porphyromonas gingivalis* (DSM-20709) were obtained from DSMZ (Deutsche Sammlung von Mikroorganismen und Zellkulturen, Braunschweig, Germany) to be used in this study. Bacteria were grown and maintained on Schaedler blood agar plates (provided by the Institute of Microbiology and Hygiene, University Medical Center Regensburg, Germany) in a microincubator (MI23NK, SCHOLZEN Microbiology Systems, St. Margrethen, Switzerland) under anaerobic conditions (80% N_2_, 10% CO_2_, and 10% H_2_). Modified Fluid Universal Medium (mFUM) supplemented with 67 mmol/L Sørensen’s buffer and containing 0.3% (w/v) glucose was employed as a basal liquid medium (Gmür and Guggenheim, 1983; Guggenheim et al., 2001). For preparation of planktonic cultures, colonies were picked and suspended in 5 mL of mFUM with 0.5 mL fetal bovine serum (FBS; Gibco^®^ Life Technologies, Carlsbad, CA, United States) overnight under anaerobic conditions. From these 1 mL was transferred to fresh 5 mL of mFUM with 0.5 mL FBS and incubated for another 6 h under anaerobic conditions to obtain bacteria in the logarithmic phase of growth. Afterwards, suspensions were harvested by centrifugation (ROTINA 420 R, Hettich Lab Technology, Tuttlingen, Germany) and resuspended in mFUM yielding an optical density (OD) of 1.0, as measured by means of a spectrophotometer at 600 nm (Ultrospec 3300 pro, Amersham Biosciences, Amersham, United Kingdom). Bacterial suspensions then were diluted 1:9 in the biofilm culture medium (BCM) consisting of 50% mFUM, 10% FBS, and 40% whole unstimulated human saliva (saliva) that had been pooled from two volunteers (authors V-SS and DM; approved by the internal review board of the University of Regensburg) and filter-sterilized (Acrodisc^®^ Syringe Filters, Pall, Newquay, United Kingdom) (Kalfas and Rundegren, 1991; Ruhl et al., 2011).

Polymicrobial Biofilms were formed in 96-well polystyrene culture plates (Corning^®^ Costar^®^, Corning, NY, United States). Firstly, wells were incubated for 2 h with saliva for simulation of pellicle-coating. After that, saliva was discarded, and wells were filled with 200 μL of BCM containing *A. naeslundii, F. nucleatum*, and *P. gingivalis* and incubated under anaerobic conditions. After 24 and 48 h, medium was carefully removed and 200 μL fresh BCM was added. For all experiments, the total culture period was 72 h.

### Antimicrobial Assay

After the total biofilm culture period of 72 h, medium was carefully discarded from the wells. Then, biofilms were either incubated with 50 μL phosphate buffered saline (PBS; Biochrom, Berlin, Germany; groups PS-L-, PS-L+) or 50 μL PS (100 μM SAPYR or SAGUA) in the dark for 20 min and then either illuminated for 10 min (groups SAPYR+L+, SAGUA+L+) or kept in the dark for 10 min (groups SAPYR+L-, SAGUA+L-). For positive controls, biofilms were incubated with CHX (0.06 and 0.2%) or MET (15 and 50 μg/mL) for a total of 30 min (50 μL each). Immediately afterwards, PBS, PS, CHX, or MET was carefully removed and the biofilm of each well was brought to suspension with 200 μL PBS and transferred to an Eppendorf tube. These were placed in an ultrasonic water-bath chamber (Sonorex Super RK 102 H, Bandelin, Berlin, Germany) obtaining a frequency of 35 kHz for 10 min and vortexed (REAX top, Heidolph Instruments, Schwabach, Germany) for 5 s to separate aggregated bacteria. Then 10-fold serial dilutions (10^-2^–10^-7^) were prepared in PBS and aliquots (180 μL) were plated on Schaedler blood agar and incubated anaerobically for 72 h. Afterwards, colony forming units (CFUs) were evaluated. Bacterial species on agar plates were discriminated by their unique colony morphology with the aid of a stereomicroscope.

### Flow Cytometric Analysis for Cytoplasmic Membrane Damage

For flow cytometry, propidium iodide (PI; Sigma-Aldrich) was used as a fluorescent dye to evaluate integrity of cytoplasmic membranes (Petit et al., 1993). Biofilms were prepared, treated and brought to suspension as described above. After that, samples were centrifuged once at 8000 rpm for 3 min and resuspended in 1 mL PBS. Then, 10 μL of each sample were mixed with 985 μL PBS and 5 μL PI (5 μg/mL), incubated for 5 min in the dark at room temperature and immediately processed by a FACSCanto flow cytometer (Becton Dickinson, Franklin Lakes, NJ, United States) equipped with a 488 nm air-cooled solid-state laser with output of 20 mW. Red fluorescence emitted by PI was detected on FL3. Bacterial cells were gated on FSC/SSC dot plots from which FL3/FSC dot plots were derived. In all cases, 10,000 events were counted.

### Spectroscopic Measurements for Release of Nucleic Acids

Damage of cytoplasmic membranes was further assessed by measuring the amount of nucleic acids released from the cytoplasm spectroscopically at 260 nm (Chen and Cooper, 2002). Biofilms were formed and treated as described above. As positive control for cytoplasmic membrane damage, biofilms were incubated with 100 μL lysozyme (40,000 units/mg; Sigma-Aldrich) for 30 min at 37°C. Then, 100 μL Proteinase K (7–14 units/mg; Sigma-Aldrich) and 200 μL sodium dodecyl sulfate (1%) were added and incubated for another 30 min at 37°C. Without discarding the supernatants, all samples were brought to suspension by adding 150 μL PBS and transferred to Eppendorf tubes. These were placed in an ultrasonic water-bath chamber (Sonorex Super RK 102 H; 35 kHz) for 10 min and centrifuged (13,000 rpm; 5 min). Afterwards, the supernatants were collected and assessed for release of nucleic acids by measuring the OD at 260 nm by means of a NanoDrop^TM^ 2000 spectrophotometer (PEQLAB, Erlangen, Germany).

### Scanning Electron Microscopy (SEM)

Biofilms were prepared on Permanox^®^ Chamber Slides (Nunc^®^ Lab-Tek^®^ Permanox^®^, 4.2 cm^2^/well, Sigma-Aldrich) and treated as described above. The samples were fixed by adding 2.5% glutaraldehyde buffered with Sørensen’s phosphate buffer (0.1 M; pH 7.4) at room temperature for 2 h. Each sample was washed twice with PBS and three times with distilled water for 15 min each. Then, the fixed samples were additionally dehydrated using 30, 50, 70, 80, 90, 96, and 100% (v/v) graded ethanol, 20 min each. After air-drying overnight in a desiccator, the growth-chambers were removed and the slides were stuck on SEM stubs (ø 25 mm). For coating, samples were purged with argon and sputtered with platinum for 30 s using a SCD 005 Sputter Coater (Bal-Tec, Balzers, Liechtenstein). Biofilms were examined using a Quanta 400 FEG scanning electron microscope (FEI Company, Hillsboro, OR, United States) in high vacuum mode at 2 kV with 6–7 mm working distance. Tilt and focus were adjusted to ensure optimum viewing. Images were taken from randomly selected fields on the slides.

### PS-Uptake Measurements

The amount of PS-uptake was measured by spectrophotometric measurement of the supernatants of bacterial cultures after incubation with the respective PS, as described earlier (Maisch et al., 2007; George et al., 2009). Planktonic cultures of *A. naeslundii, F. nucleatum*, and *P. gingivalis* were grown overnight as described above and adjusted to an OD of 1.0. Then, these suspensions were incubated in the dark for 20 min with 100 μM SAPYR or with 100 μM Methylene Blue (Sigma-Aldrich, St. Louis, MO, United States), which acted as a positive control. After incubation, cells were centrifuged (13,000 rpm; 5 min) and the supernatants were measured spectrophotometrically (SPECORD^®^ 50 PLUS, Analytik Jena, Jena, Germany) to record absorption of PS not bound to bacterial cells. Bacteria without PS and PS-solutions served as controls. This experiment was repeated thrice.

### Data Analysis

All CFU results are shown as medians, 1st and 3rd quartiles and were calculated using SPSS for Windows, version 25 (SPSS Inc., Chicago, IL, United States) from the values of at least six independent experiments, each performed in duplicate. Horizontal solid and dashed lines represent reductions of 3 and 5 log_10_ steps of CFU, respectively, compared to the untreated control group PS-L-. Medians on or below these lines demonstrate an antimicrobial efficacy of 99.9% (3 log_10_) or 99.999% (5 log_10_), at least, which is declared as biologically relevant antimicrobial activity or disinfectant effect according to guidelines of infection control (Boyce and Pittet, 2002).

Flow cytometric data were analyzed using FACSDiva^TM^ software, version 5.0.2 (Becton Dickinson). The percentages of unstained and PI-stained bacteria were calculated as medians, 1st and 3rd quartiles using SPSS from the values of six independent experiments, each performed in duplicate.

Results from the spectroscopic measurements for release of nucleic acids are shown as medians, 1st and 3rd quartiles, calculated from the values of seven independent experiments using SPSS.

## Results

## Antimicrobial Assay

**FIGURE 2:**
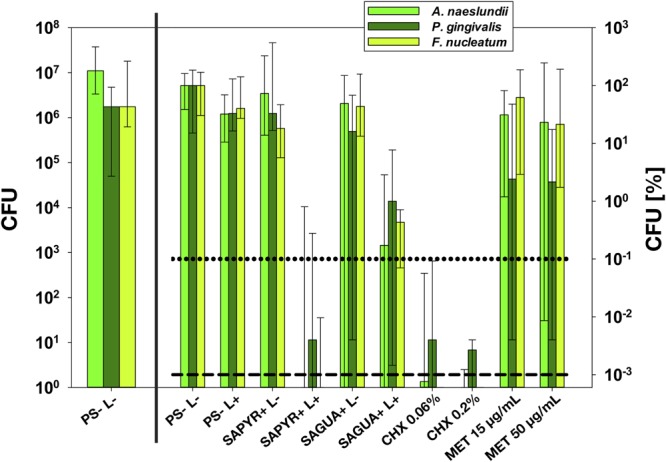
Antimicrobial assay. All results are depicted as medians, 1st and 3rd quartiles from six independent experiments in duplicates on a log_10_-scaled ordinate. **(Left)** shows untreated control group PS-L– as absolute colony forming units (CFU) values. **(Right)** shows relative CFU data (CFU [%]) with untreated control group (PS-L–) set to 100% for each bacterial strain. Horizontal dotted and dashed lines represent CFU-reductions of 3 log_10_ and 5 log_10_, respectively.

Untreated biofilms (PS-L-) showed a slightly higher growth of *A. naeslundii* (1.1 × 10^7^) of about 1 log_10_-step as compared to *F. nucleatum* and *P. gingivalis* (both: 1.8 × 10^6^), as shown as absolute CFU values in the left panel of **Figure 2**. The right panel of **Figure 2** depicts relative CFU data (CFU [%]) with untreated controls (groups PS-L-) set to 100% separately for each bacterial strain.

Antimicrobial photodynamic therapy with SAPYR reduced CFU of *A. naeslundii* and *F. nucleatum* by 6.0 and 6.1 log_10_ and *P. gingivalis* by 4.4 log_10_. In contrast, aPDT with SAGUA led to reductions of 2.8 log_10_ against *A. naeslundii*, 2.4 log_10_ against *F. nucleatum* and 2.0 log_10_ against *P. gingivalis* only. In all cases, there was no effect of treatment with PS or light only.

Chlorhexidine 0.06 and 0.2% led to reductions of 4.4–6.1 log_10_ or 4.6–6.1 log_10_, respectively, with *P. gingivalis* representing the less susceptible species. In contrast, *P. gingivalis* was the only species that could be inactivated by treatment with MET by >0.5 log_10_ (1.6 or 1.8 log_10_ at concentrations of 15 μg/mL or 50 μg/mL, respectively).

### Flow Cytometric Analysis for Cytoplasmic Membrane Damage

**FIGURE 3:**
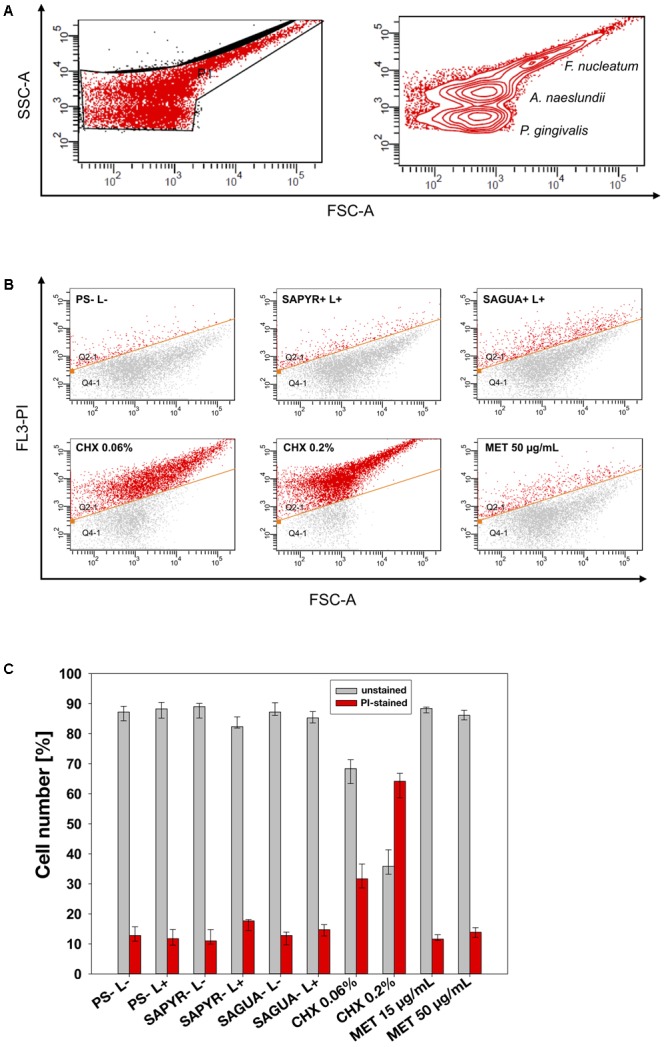
Flow cytometric analysis for cytoplasmic membrane damage. **(A)** Bacterial cell populations gated on dot plot FSC vs. SSC with P1 showing the chosen region of interest (ROI). Three populations could be discriminated (circles show high number of reads). **(B)** Exemplary dot plots for untreated controls (PS-L–) and groups treated with aPDT (SAPYR+L+; SAGUA+L+), CHX (CHX 0.06%; CHX 0.2%) or MET (MET 50 μg/mL), respectively. The gate below the orange line indicates unstained cells with intact membrane, while the gate above the orange line indicates PI-stained cells with damaged membrane. **(C)** Summarized median percentages, 1st and 3rd quartiles of unstained (gray) as well as PI-stained bacterial cells (red) are shown for all experimental groups. Membrane damage could only be observed after treatment with CHX in a concentration-dependent manner.

Flow cytometry with PI as fluorescent dye was employed to evaluate damage of the cytoplasmic membrane as cells with intact membranes are not permeable to PI (Petit et al., 1993). When determining the region of interest (ROI) in untreated controls, three cell populations indicative for the three bacterial species could be clearly determined on dot plots FSC vs. SSC depending on the respective size and granularity of the bacterial cells (**Figure 3A**).

Exemplary dot plots are shown in **Figure 3B** for selected groups. **Figure 3C** shows summarized percentages of unstained as well as PI-stained bacterial cells for all groups. Untreated biofilms (PS-L-) showed a proportion of PI-positive cells of 13%. Neither treatment with PS or light alone, nor aPDT with SAPYR and SAGUA nor MET at both concentrations led to an increase of PI-positive cells as compared to untreated controls (median of PI-positive cells: 11–15%). In contrast, treatment with CHX led to a concentration-dependent median increase of PI-positive cells to 32% (CHX 0.06%) or 64% (CHX 0.2%), respectively.

### Spectroscopic Measurements for Release of Nucleic Acids

**FIGURE 4:**
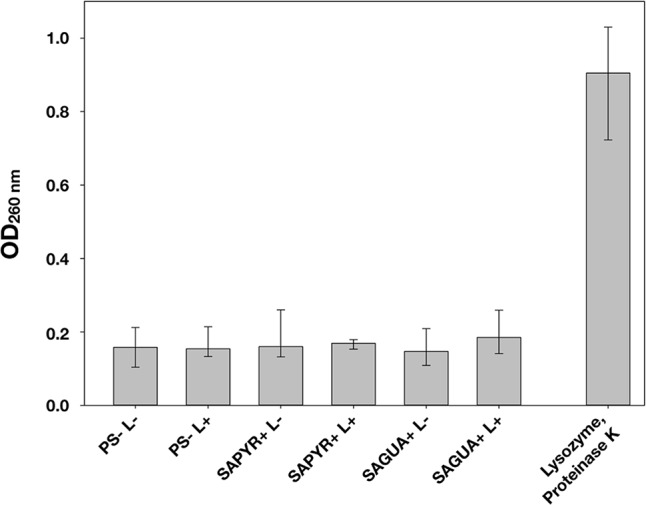
Spectroscopic measurements for release of nucleic acids. OD medians, 1st and 3rd quartiles of the supernatants of biofilms treated with phenalen-1-one mediated aPDT (groups: PS-L–, PS-L+, SAPYR+L-, SAPYR+L+, SAGUA+L-, and SAGUA+L+) or positive control (lysozyme treatment followed by Proteinase K digestion), as measured at 260 nm for release of nucleic acids.

Damage of cytoplasmic membranes was further assessed by measuring the release of nucleic acids spectroscopically at 260 nm. As compared to the untreated control group (PS-L-), neither treatment with light or PS alone nor aPDT with SAPYR or SAGUA led to an increase in OD at 260 nm (median OD values ranging from 0.15 to 0.19). In contrast, in the positive control group comprising lysis with lysozyme following Proteinase K digestion a median OD of 0.91 was found (**Figure 4**).

### Scanning Electron Microscopy (SEM)

**FIGURE 5:**
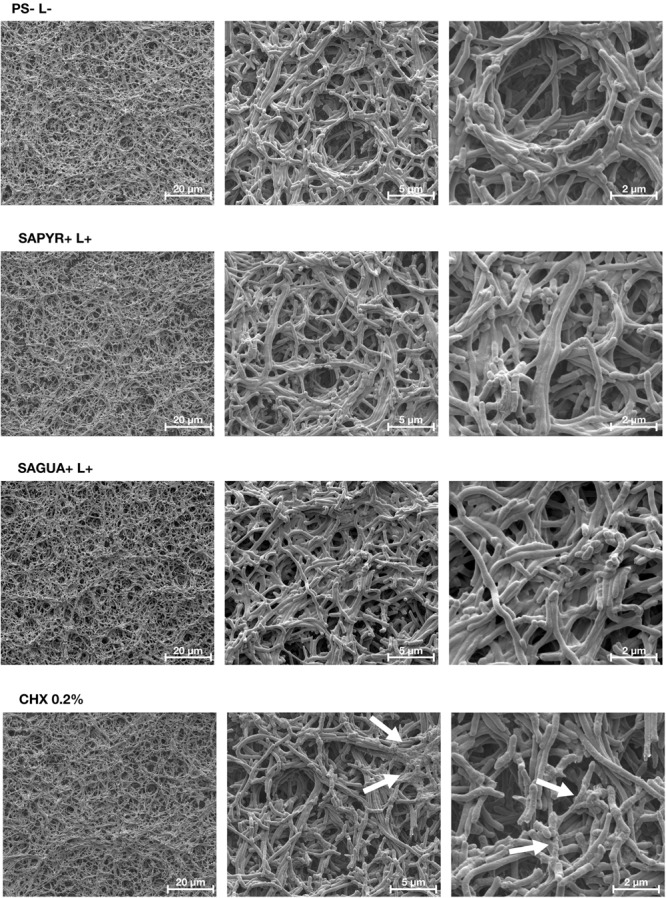
Exemplary visualization of polymicrobial biofilms by means of scanning electron microscopy. Exemplary SEM visualization of randomly selected fields of untreated biofilms (PS-L–), biofilms treated with aPDT using SAPYR (SAPYR+L+) or SAGUA (SAGUA+L+) or treated with CHX (CHX 0.2%) in 3,000-fold, 12,000-fold, and 24,000-fold magnification. In the CHX-treated biofilms white arrows show debris most likely originating from killed cells on the top layer of the biofilms.

**Figure 5** shows exemplary SEM images taken from randomly selected fields of untreated biofilms (group PS-L-) and biofilms treated with aPDT with SAPYR (SAPYR+L+) or SAGUA (SAGUA+L+) or CHX (CHX 0.2%). Untreated biofilms showed a multi-layered biofilm structure with typical voids. While aPDT with SAPYR resulted in no visible impact on overall biofilm structure or bacterial cell morphology, in CHX-treated samples debris most likely from killed cells was detectable on the biofilm surface.

### PS-Uptake Measurements

**FIGURE 6:**
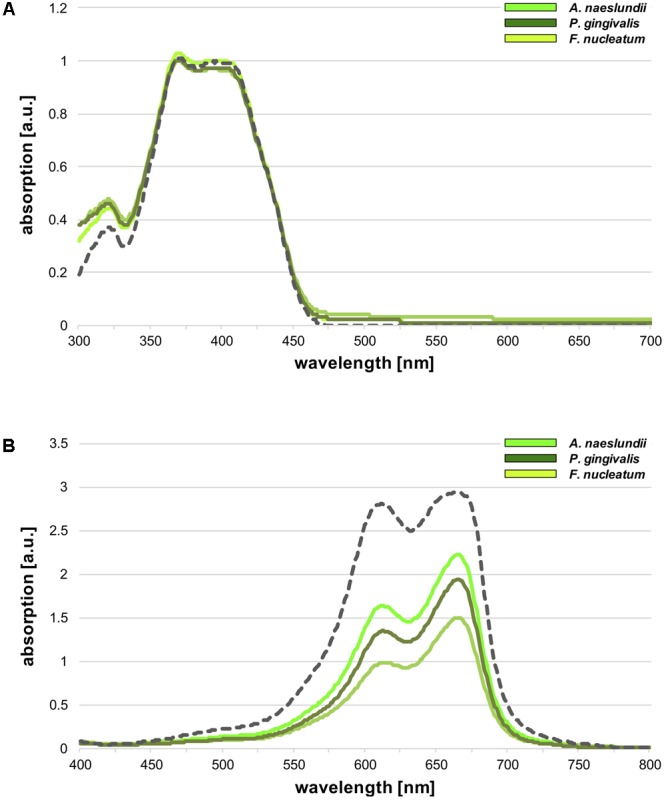
Photosensitizers (PS)-uptake measurements. Spectrophotometrical evaluation of PS-uptake for **(A)** SAPYR and **(B)** Methylene Blue. Gray dotted lines show absorption spectra of the PS solutions (100 μM), while green solid lines show absorption spectra of the supernatants of bacteria incubated with the respective PS (neon green: *Actinomyces naeslundii*; yellowish green: *Fusobacterium nucleatum*; dark green: *Porphyromonas gingivalis*).

The amount of PS-uptake was determined spectrophotometrically for SAPYR and Methylene Blue by measuring the absorption of the respective PS remaining in the supernatants of planktonic cultures after incubation for 30 min. **Figure 6** shows absorption spectra for SAPYR and the supernatants of bacteria incubated with SAPYR (**Figure 6A**) or Methylene Blue and the supernatants of bacteria incubated with Methylene Blue (**Figure 6B**), respectively. For Methylene Blue, there was a clear decrease in absorption in the supernatants as compared to the PS-suspension indicating PS-uptake or strong attachment to the bacterial cells. In contrast, for SAPYR no uptake or attachment of the PS to the bacterial cells was found as shown by the absent decrease in absorption in the supernatants.

## Discussion

Traditionally, culture-based methods (like CFU assays) have been employed for evaluating the efficacy of given antimicrobial approaches. Hereby, “death” of bacterial cells is defined by their inability to replicate and form colonies on solid agar medium (Berney et al., 2007). This means that the effect of an antimicrobial approach can only be evaluated retrospectively after a given incubation period as several cell divisions are needed for forming a visible colony on agar (Nebe-von Caron et al., 1998). CFU assays facilitate standardization of results from distinct studies, as the calculated log_10_ reduction rates can easily be compared. On the other hand, this technique does not permit insights to the mechanisms of action of given antimicrobials. In this instance, a complementary technique may be flow cytometry which allows for measurements of different vital parameters within a cell when combined with appropriate fluorescent dyes (Novo et al., 2000; Kennedy et al., 2011). This may be especially valuable in narrowing down to the mechanism of novel antimicrobial approaches, such as aPDT, and revealing their main target structures. As for phenalen-1-one-mediated aPDT incubation periods in the seconds range are sufficient to reach disinfecting effects (≥5 log_10_ steps reduction of CFU) against planktonic bacteria irrespective of their Gram-staining characteristics (Späth et al., 2014; Tabenski et al., 2016; Muehler et al., 2017), we proposed that damage of cytoplasmic membranes rather than of DNA may be the main mechanism of aPDT with this class of PS. Therefore, in the present study we combined traditional culture techniques and flow cytometry to investigate the antimicrobial efficacy of phenalen-1-one-mediated aPDT when applied to biofilms formed by three putative periodontal pathogens *in vitro* in order to get first insights in its mechanism of action by evaluating the damage of cytoplasmic membranes.

For this purpose, a polymicrobial biofilm was established *in vitro* from *Actinomyces naeslundii, Fusobacterium nucleatum*, and *Porphyromonas gingivalis*, whereby culture conditions were derived and slightly modified from the well-known Zurich biofilm model (Guggenheim et al., 2001; Belibasakis and Thurnheer, 2014). In this model, *A. naeslundii* represents an essential early colonizer (Dige et al., 2009), while *F. nucleatum* is known to act as a bridge between early and late colonizers (Bolstad et al., 1996) and *P. gingivalis* is a late colonizer and keystone pathogen promoting the formation of dysbiotic periodontitis-associated microbial communities (Hajishengallis and Lamont, 2016). In contrast to the original Zurich biofilm model, biofilms were not cultured on hydroxyapatite disks, but in flat-bottomed 96-well plates to allow for high-throughput screening and, particularly, for facilitating standardized irradiation procedures which would be compromised with biofilms forming on both sides of the hydroxyapatite disks. Exemplary SEM visualization of untreated biofilms revealed a multi-layered biofilm architecture with typical voids, similarly to naturally occurring oral biofilms (Rudney et al., 2012). As positive control for antimicrobial efficacy, CHX was used as gold-standard antiseptic in typical concentrations applied in oral care (0.06% as well as 0.2%). Further, MET was included as standard antibiotic in clinical periodontal practice for inactivation of anaerobes (Slots, 2004). Here, the concentration of 15 μg/mL was chosen as it can be typically reached in plasma, gingival crevicular fluid and saliva after systemic administration (Pähkla et al., 2005; Belibasakis and Thurnheer, 2014), while 50 μg/mL was additionally used as slightly higher concentration.

Treatment with CHX resulted in reductions of about 6 log_10_ against *A. naeslundii* and *F. nucleatum* and about 4.5 log_10_ against *P. gingivalis* irrespective of the applied concentration. Identical antimicrobial efficacy was found for aPDT employing SAPYR as a PS. This is noteworthy because despite equal total treatment periods (30 min) for CHX and aPDT, the period of actual antimicrobial activity of aPDT is limited to the period of light-activation (here: 10 min), while the preceding pre-incubation period (here: 20 min) just ensures sufficient penetration of the PS throughout the extracellular polymeric substance (EPS) of the biofilm (Mah and O’Toole, 2001). The antimicrobial photodynamic efficacy of SAGUA (2.0–2.8 log_10_) was clearly inferior as compared to SAPYR (4.4–6.1 log_10_) although the opposite was found in a previous study for inactivation of planktonic bacteria (Tabenski et al., 2016). This may be explained by the nature of the positively charged guanidinium group of SAGUA which can establish hydrogen bonds with phosphate and carboxylate groups (Tabenski et al., 2016). This may improve the attachment of SAGUA toward bacterial cell walls in planktonic cultures, but otherwise hamper diffusion of SAGUA through the EPS of a biofilm by interacting with negatively charged EPS molecules. Another point may be the inferior singlet oxygen quantum yield Φ_Δ_ of SAGUA as compared to SAPYR (0.86 vs. 0.99) (Tabenski et al., 2016).

MET reduced only CFU of *P. gingivalis* in a concentration-dependent manner by 1.6 log_10_ or 1.8 log_10_, while *A. naeslundii* and *F. nucleatum* were not affected at all. This is in line with literature data, where Belibasakis and Thurnheer (2014) found no CFU-reductions after applying 15 μg/mL MET even for an incubation period of 24 h in the Zurich subgingival biofilm model. Furthermore, Wang et al. (2015) reported a minimum biofilm eradication concentration of 800 μg/mL MET for a double-species *in vitro* biofilm of *F. nucleatum* and *P. gingivalis* after treatment for 24 h. Despite *in vitro* data, this is further evidence that any administration of antibiotics in clinical periodontal treatment is obsolete without mechanical disruption of the subgingival biofilms (Belibasakis and Thurnheer, 2014). Accordingly, minimum bactericidal concentrations of MET are in a much lower range for planktonic cultures of periodontal pathogens [e.g., 25 and 10 μg/mL for *F. nucleatum* or *P. gingivalis*, respectively (Wang et al., 2015)], which is clinically achievable in saliva and gingival crevicular fluid (Pähkla et al., 2005).

Although there are some studies that utilized flow cytometry as a simple measure for antimicrobial efficacy of aPDT (Bulit et al., 2014; Manoil et al., 2014; Marinic et al., 2015), the present study is the first one employing flow cytometry for getting insights into the mechanism of action of aPDT. We employed PI, a positively charged nucleic acid stain that is only able to enter bacterial cells through permeabilized membranes, in order to assess cytoplasmic membrane damage (Petit et al., 1993; Joux and Lebaron, 2000). Here, CHX served as a positive control because it is well known to damage the bacterial cytoplasmic membrane followed by leakage of cytoplasmic constituents (McDonnell and Russell, 1999). MET served as a negative control as its antimicrobial effect is based on inducing DNA strand breaks, but not on cytoplasmic membrane damage (Edwards, 1979).

In the chosen ROI, three cell populations could be clearly discriminated according to the respective size and granularity of the bacterial cells. As the small size of bacteria (especially of *P. gingivalis*) complicates distinguishing between small bacterial cells and abiotic cellular debris (Ambriz-Aviña et al., 2014), the correctness of the chosen ROI was confirmed by additional nucleic acid staining with SYBR green (data not shown). The populations indicative for *F. nucleatum* and *P. gingivalis* and the shape of their respective dot plots are in accordance to those recently reported by Ciandrini et al. (2014) for single-species biofilms of these species.

As expected, flow cytometry revealed a clear and concentration-dependent increase in the percentage of PI-positive cells after treatment with CHX and no increase after treatment with MET. At first glance, a proportion of 64% PI-positive cells after treatment with 0.2% CHX seems not matching a reduction of 4.6–6.1 log_10_ steps of CFU, but this phenomenon just shows that very distinct vital parameters, i.e., integrity of cytoplasmic membranes or ability of bacterial cells to replicate, are measured by means of these techniques. Therefore, a bacterial cell with not completely permeabilized cytoplasmic membrane may still not be able to replicate due to the total incurred extent of damage. This phenomenon is known as “viable, but not culturable” (VBNC) state (Joux and Lebaron, 2000), in which bacteria usually show decreased metabolic activity and little or no ability to replicate (Tamburini et al., 2014). In contrast to CHX, aPDT led to no increase in PI-positive cells neither when employing SAPYR nor SAGUA.

For further assessment of cytoplasmic membrane damage after aPDT with SAPYR or SAGUA, release of nucleic acids after treatment was measured spectroscopically at 260 nm, which has been described to serve as a reliable indicator for assessing membrane integrity (Chen and Cooper, 2002). As CHX was not applicable for these experiments due to its characteristic absorption maximum at 260 nm (Hiraishi et al., 2008), a lysis step with lysozyme followed by Proteinase K digestion served as positive control for cytoplasmic membrane damage. In accordance with the results from flow cytometry, there was no increased release of nucleic acids after aPDT with SAPYR or SAGUA as compared to the untreated control group. These findings are in line with the exemplary SEM visualizations, where no impact on overall biofilm structure or bacterial cell morphology could be detected after treatment with aPDT using SAPYR or SAGUA as PS. In contrast, in CHX-treated samples debris was visible on the biofilm surface, most likely originating from disrupted cells (Wang et al., 2017).

However, these results do not necessarily implicate that cytoplasmic membranes are not targeted at all by phenalen-1-one-mediated aPDT, as it is usually assumed that permeability to “exclusion dyes” like PI (molecular weight 668.4 g/mol) is associated with large and irreparable gaps in the cytoplasmic membrane (Novo et al., 2000; Falcioni et al., 2008). Likewise, leakage of large molecules like nucleic acids occurs only after pronounced damage of cytoplasmic membranes, while small ions like potassium or phosphate tend to leach out earlier (Chen and Cooper, 2002). Consequently, the oxidative burst induced by aPDT potentially may not lead to these large gaps in the cytoplasmic membranes, but to a depolarization of the membrane potential only.

For evaluating the cellular localization of SAPYR, PS-uptake was measured spectrophotometrically. Here, Methylene Blue served as a positive control because this PS seems to locate intracellularly as its photodynamic efficacy can be enhanced by addition of efflux pump inhibitors (Tegos et al., 2008). Accordingly, a clear decrease in absorption could be found in the supernatants of bacterial cells after incubation with Methylene Blue, indicating cellular uptake or strong attachment to the bacteria. In contrast, the supernatants of bacterial cells incubated with SAPYR exhibited no decrease in absorption at all. Therefore, cellular uptake of SAPYR seems very improbable. As it is known that a PS has to build up high intracellular concentrations for resulting in DNA damage upon irradiation (Cadet et al., 2010), DNA may not be the target structure of aPDT with SAPYR. It is rather plausible that SAPYR is just electrostatically attracted to bacterial cell surfaces by its positive charge but does not directly attach toward these cell surfaces.

This study represents a first step in investigating the bacterial target structures of the oxidative burst mediated by aPDT with phenalene-1-one derivatives. For deeper insights into the mechanism and damage patterns of aPDT, future studies must combine distinct methods for resolving these complex mechanisms. For example, so-called multi-parameter flow cytometry employing many fluorescent markers for distinct vital parameters may represent a valuable approach for assessing the effects of aPDT on distinct cellular structures (Novo et al., 2000; Ambriz-Aviña et al., 2014).

Furthermore, potential harmful effects on mammalian tissues must be precluded before phenalen-1-one-mediated aPDT can be applied clinically. In a recent study, aPDT with various phenalen-1-one derivatives was investigated for its antimicrobial efficacy toward dermal pathogens and for its eukaryotic toxicity toward keratinocytes as compared to the biocide benzalkonium chloride for assessing potential effective concentration ranges (i.e., ≥5 log_10_ reduction of CFU while ≥80% survival of keratinocytes). For aPDT with SAPYR a broad effective concentration range was found, while for benzalkonium chloride there was no effective concentration range at all (Muehler et al., 2017). These encouraging results must now be corroborated in further biocompatibility studies.

## Conclusion

• Antimicrobial photodynamic therapy with SAPYR is as effective as CHX 0.2% when applied to an *in vitro* biofilm comprising *A. naeslundii, F. nucleatum*, and *P. gingivalis* leading to reduction rates of 4.4–6.1 log_10_ steps while aPDT with SAGUA is less effective.
• In contrast to CHX, phenalen-1-one-mediated aPDT leads to no damage of cytoplasmic membranes as revealed by flow cytometry using PI as a fluorescent dye. Likewise, no release of nucleic acids could be detected after aPDT with SAPYR or SAGUA.
• SAPYR exhibits neither uptake nor strong attachment toward bacterial cells as opposed to Methylene Blue.

## Author Contributions

FC, TM, and WB conceived and designed the experiments. V-SS and DM performed the experiments. TT and GB helped with setting up the biofilm model. FC, V-SS, DM, K-AH, TM, and WB analyzed the data. FC wrote the manuscript with input from all the other authors. All authors reviewed the manuscript.

## Conflict of Interest Statement

The authors declare that the research was conducted in the absence of any commercial or financial relationships that could be construed as a potential conflict of interest. The reviewer TR and handling Editor declared their shared affiliation.
